# Surface Display and Bioactivity of *Bombyx mori* Acetylcholinesterase on *Pichia pastoris*

**DOI:** 10.1371/journal.pone.0070451

**Published:** 2013-08-05

**Authors:** Jie-Xian Dong, Xi Xie, Yong-Sheng He, Ross C. Beier, Yuan-Ming Sun, Zhen-Lin Xu, Wei-Jian Wu, Yu-Dong Shen, Zhi-Li Xiao, Li-Na Lai, Hong Wang, Jin-Yi Yang

**Affiliations:** 1 Guangdong Provincial Key Laboratory of Food Quality and Safety, South China Agricultural University, Guangzhou, Guangdong Province, China; 2 Shenzhen Academy of Metrology and Quality Inspection, Shenzhen, Guangdong Province, China; 3 United States Department of Agriculture, Agricultural Research Service, Southern Plains Agricultural Research Center, Food and Feed Safety Research Unit, College Station, Texas, United States of America; Weizmann Institute of Science, Israel

## Abstract

A *Pichia pastoris* (*P. pastoris*) cell surface display system of *Bombyx mori* acetylcholinesterase (*Bm*AChE) was constructed and its bioactivity was studied. The modified *Bombyx mori* acetylcholinesterase gene (*bmace*) was fused with the anchor protein (*AGα1)* from *Saccharomyces cerevisiae* and transformed into *P. pastoris* strain GS115. The recombinant strain harboring the fusion gene *bmace*-*AGα1* was induced to display *Bm*AChE on the *P. pastoris* cell surface. Fluorescence microscopy and flow cytometry assays revealed that the *Bm*AChE was successfully displayed on the cell surface of *P. pastoris* GS115. The enzyme activity of the displayed *Bm*AChE was detected by the Ellman method at 787.7 U/g (wet cell weight). In addition, bioactivity of the displayed *Bm*AChE was verified by inhibition tests conducted with eserine, and with carbamate and organophosphorus pesticides. The displayed *Bm*AChE had an IC_50_ of 4.17×10^−8^ M and was highly sensitive to eserine and five carbamate pesticides, as well as seven organophosphorus pesticides. Results suggest that the displayed *Bm*AChE had good bioactivity.

## Introduction

The intensive use of carbamate (CB) and organophosphorus (OP) pesticides in recent years has led to potentially dangerous effects on human and animal health. The control of pesticide residues in food and the environment is of great importance to minimize the risk to consumers and environmental animal species. Routinely, CB and OP pesticide residues are measured by instrumental methods, such as gas chromatography, liquid chromatography and gas chromatography–tandem mass spectrometry [1], [2], [3]. There is a growing interest in more rapid and low-cost field-portable detection systems. A promising approach involves the use of screening enzyme-linked immunoassays [4]. However, these assays require broad-specificity antibodies that are difficult to develop. Nevertheless, an enzyme-based method was demonstrated to be an efficient and rapid method for the detection of pesticides because it was inexpensive, allowed high sample throughput, and was easily adapted for use in Asian markets [5].

Previously, acetylcholinesterase (AChE), aldehyde dehydrogenase, alkaline and acid phosphatase, butyrylcholinesterase, organophosphorus hydrolase and tyrosinase have been investigated for their ability to detect pesticides in water and other matrices such as soil, food and beverages [6]. However, AChE has been most often used for enzymatic detection of pesticides because of its broad substrate specificity and good sensitivity [6].

AChE is a key enzyme in the cholinergic system that regulates the level of acetylcholine and terminates nerve impulses by catalyzing the hydrolysis of the neurotransmitter acetylcholine in the synaptic cleft [7], [8]. The enzyme activity of AChE can be inhibited by CB and OP pesticides. Therefore, it is feasible to use AChE for the detection of CB and OP pesticides based on the degree of AChE activity inhibition [9]. AChE has been isolated by traditional extraction methods from natural tissues [10], [11], [12] or from secretions of engineered cells [8], [13]. Isolation from these areas requires an enzyme purification step, which leads to higher preparation costs. However, natively displayed molecules on the surface of cells presents another option, which is currently of great interest. Many heterologous proteins and polypeptides have been displayed on the surface of cells, and these displays have been widely used [14], [15], [16], [17], [18]. The use of displayed molecules on the cell surface can save tedious purification steps required for enzymes used in traditional immobilization methods. Further, protein engineering can help generate a surface display of enzymes that can be used in efficient high-throughput screening methods for residue detection. In cell surface display development, the anchor protein is a necessary component. The most frequently used anchor is the *N*-terminal fusion display of α-agglutinin from *Saccharomyces cerevisiae* (*S. cerevisiae*), which is composed of a secretion-signal region, an active region, a serine- and threonine-rich support region, and a putative glycosylphosphatidylinositol anchor-attachment protein. AGα1 protein is one of the α-agglutinins in *N*-terminal fusion displays [19]. Different enzymes used for the detection of pesticides, like organophosphorus hydrolase [20] and mouse AChE [21], have been expressed on the surface of microorganisms. In this study we took advantage of the domestic silkworm, *Bombyx mori* as the *ace* gene source to investigate the sensitivity of the displayed AChE for pesticides. Domesticated silkworms have not suffered from pesticide selection, severe competition for food, or from finding good mating partners over the last number of decades. As a result, the sensitivity of AChE in domestic silkworms would be expected to be preserved and be more sensitive than the enzyme from the wild-type source [22], suggesting that AChE from *Bombyx mori* may be a remarkable reagent for pesticide detection.

This study was conducted with the aim to construct a cell surface display system for *Bombyx mori* AChE (*Bm*AChE). The work may lay the foundation for further sensitivity improvement by developing a displayed AChE system through recombinant molecular methods and application of whole-cell biosensors for the broad-specificity detection of CB and OP pesticides. Here we displayed the *Bm*AChE on *Pichia pastoris* (*P. pastoris*) for the first time. We cloned the AChE gene from *Bombyx mori* and the anchor protein gene *AGα1* from *Saccharomyces cerevisiae* (*S. cerevisiae*). We then constructed a stable *P. pastoris* cell surface display system for the recombinant *Bm*AChE. The display of the recombinant enzyme on the surface of the yeast was then used for the detection of CB and OP pesticides, which resulted in the development of a rapid, easy and sensitive analytical method useful for the detection of pesticide residues.

## Materials and Methods

### Strains and Media

*Escherichia coli* (*E. coli*) DH5α stored in our laboratory was used as the host for recombinant DNA manipulation. The *P. pastoris* GS115 strains and the integrative expression vector (pPIC9K) were obtained from Invitrogen Biotechnology Co. (Shanghai, China).

*E. coli* was grown in Luria-Bertani medium (1% peptone, 0.5% yeast extract, and 1% sodium chloride). *Pichia pastoris* was cultivated in yeast peptone dextrose medium (1% yeast extract, 2% peptone, and 2% glucose), and *P. pastoris* transformants were cultivated on minimal dextrose medium (MD) plates (2% glucose, 0.00004% biotin, 1.34% yeast nitrogen base (YNB) and 1.8% agarose).

### Reagents

Gel extraction kits were obtained from Tiangen (Beijing, China). Yeast genome extraction kits were obtained from Beijing ComWin Biotech Co., Ltd (Beijing, China). PrimerSTAR DNA polymerase, restriction enzymes, and dNTP were obtained from Takara Biotechnology Co. Ltd. (Dalian, China). Primers were synthesized by Shanghai Sangon Biotechnology (Shanghai, China). The mouse anti-FLAG monoclonal antibody, Alex Fluor 488 labeled goat anti-mouse IgG, acetylthiocholine iodide (ATC) and 5,5′-dithiobis(2-nitrobenzoic acid) (DTNB) were obtained from Sigma-Aldrich (St. Louis, MO, USA). CB and OP standards were obtained from the National Center of Standard Material (Beijing, China).

### Cloning and Assembly of the *bmace-AGα1* Gene

**Figure 1:**
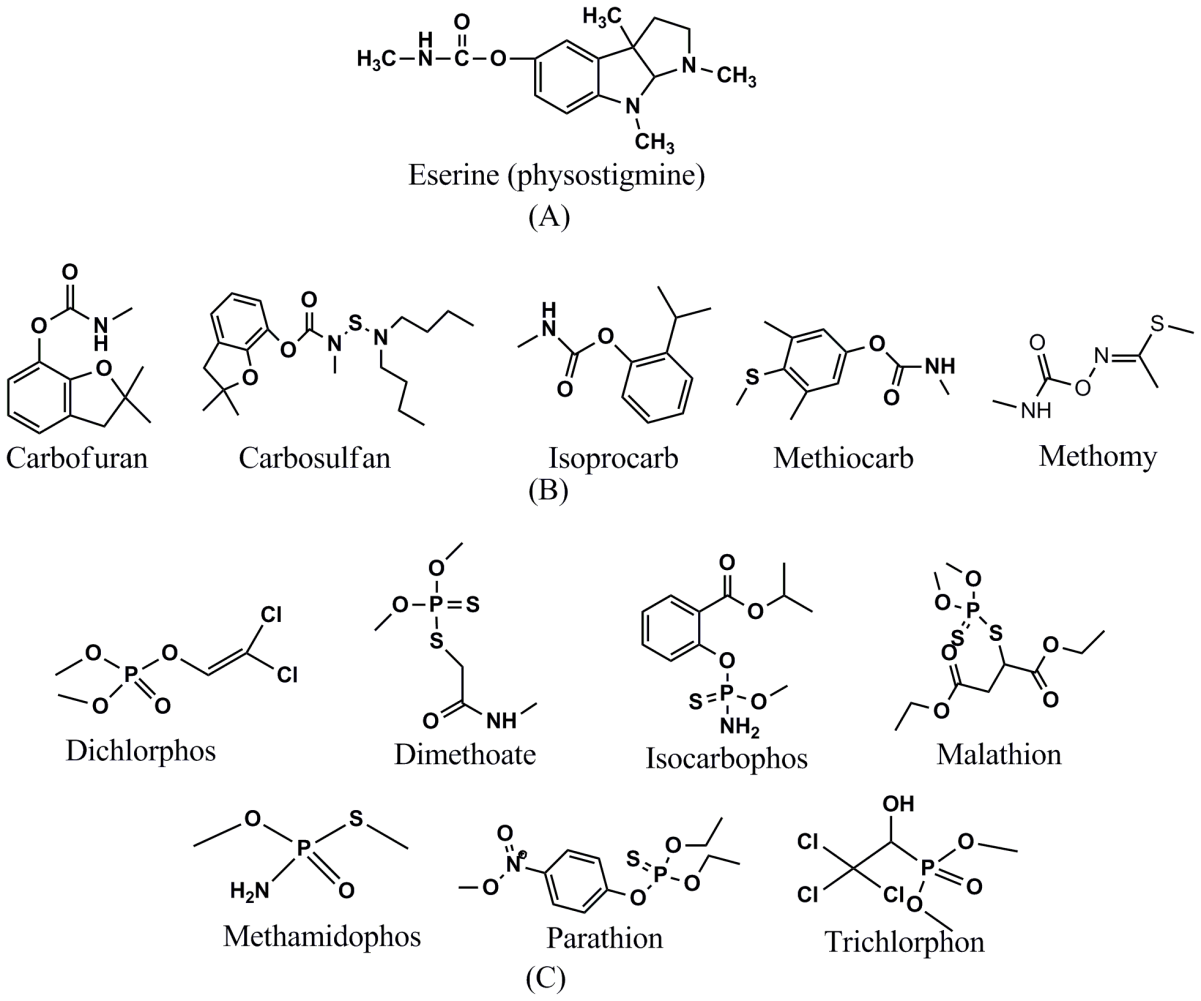
The chemical structures of the different compounds tested. (A) structure of the natural inhibitor of AChE, (B) structures of the CB pesticides, (C) structures of the OP pesticides.

The construction scheme for the plasmid containing the *bmace-AGα1* fusion gene is shown in Fig. 1; DNA fragments encoding for *Bm*AChE were amplified with the constructed vector pPIC9K–*bmace* [23] as a template without the signal peptides and the hydrophobic amino acid tail gene. The PCR process was performed using PrimerSTAR DNA Polymerase and the amplification experiment was run at a melting temperature of 94°C for 1 min, annealing at 58°C for 1 min, and extension at 72°C for 2 min, with a 30 cycle repeat. Primers used for PCR amplification containing the FLAG tag at 5′ and partial linker at 3′ were the two oligonucleotides F1 and R1, respectively (Table 1). The genome of *S. cerevisiae* was extracted using the yeast genome extraction kit, and the *AGα1* gene was amplified using the genome as template and the F2 and R2 primers listed in Table 1. The purified *bmace* and *AGα1* DNA segments (50 ng each) were spliced using overlap extension PCR to assemble the *bmace-AGα1* gene with the (Gly_4_Ser)_3_ linker. Then, the *bmace-AGα1* gene was amplified using the F1 and R2 primers (Table 1). The PCR amplification products were purified by an agarose gel DNA purification kit and stored at −20°C.

**Table 1:** Primers used for cloning *bmace* and *AGα1* genes and generating the synthetic gene encoding *bmace-AGα1* fusion protein.

F1:	5′-GCGACGCGTGACTACAAGGACGATGACGATAAGCGATCTTGGGCCAATCAC-3′
R1:	5′- CCAGAGCCACCTCCGCCTGAACCGCCTCCACCACTGCTGTAAGGACCGGT-3′
F2:	5′ -AGGCGGAGGTGGCTCTGGCGGTGGCGGATCGAACCTCGGTACAGCTAGC-3′
R2:	5′-GCCGCGGCCGCGAATAGCAGGTACGACAAAAG-3′
AOX-F	5′-GGCAAATGGCATTCTGACATCCT-3′
AOX-R	5′-GGCAAATGGCATTCTGACATCC-3′.

### Construction of the Plasmid for Cell Surface Display

The resulting PCR products from the above step were digested with the restriction enzymes *Mlu* I and *Not* I, and then ligated into the expression vector pPIC9K. The ligated products were transformed into competent *E. coli* DH5α cells for propagation of the recombinant plasmid. The recombinant plasmid pPIC9K-*bmace-AGα1* was confirmed by restriction enzyme digestion and DNA sequencing.

### *P. pastoris* Transformation and Selection

Linearized vectors were transformed into *P. pastoris* as previously described [24]. Transformed cells were spread on MD plates and incubated at 30°C for 3 d to select His^+^ transformants. Genomic integration was confirmed by performing PCR on genomic DNA with the AOX-F and AOX-R primers (Table 1).

### Expression of the *bmace-AGα1* Gene

The recombinant *P. pastoris* clone was grown in 20 mL BMGY medium (1% yeast extract, 2% peptone, 1.34% YNB, 0.00004% biotin, 1% glycerol and 100 mM potassium phosphate (pH 6.0)) in shake culture at 30°C for 24 h until the OD_600_ reached a value of more than 4. The culture (5 mL) was centrifuged at 3000×g for 5 min. The cells were induced by re-suspension with 20 mL BMMY medium (1% yeast extract, 2% peptone, 1.34% YNB, 0.00004% biotin, 0.5% methanol and 100 mM potassium phosphate (pH 6.0)) and the resulting OD_600_ was approximately 1. The induction was continued at 28°C for 4 more d by adding 200 µL of 100% methanol to the cultures daily.

### Characterization of the Displayed *Bm*AChE by Fluorescence Microscopy

The immunofluorescent labeling of yeast cells was carried out as follows: A cell suspension was centrifuged at 8000×g for 1 min, and the collected cells were washed three times with 0.01 M phosphate buffered saline (PBS) (8.0 g/L NaCl, 1.44 g/L Na_2_HPO_4_•12H_2_O, 0.2 g/L KCl, 0.24 g/L KH_2_PO_4_ (pH 7.4)). The cells were suspended and blocked with PBS containing 1% bovine serum albumin (BSA) for 0.5 h (OD_600_ = 1.0). Anti-FLAG IgG (1 µL) was added to the 200 µL cell suspension and incubated at room temperature for 1.5 h. The cells were then washed with PBS, centrifuged at 6000×g for 10 min at room temperature, suspended in 200 µL of PBS with 1 µL Alexa Fluor TM 488-conjugated goat anti-mouse IgG (1∶200), and then incubated at room temperature for 1.5 h. The PBS washed cells were observed under a fluorescence microscope (Nikon Eclipse 80i, Tokyo, Japan). The excitation and emission wavelengths used were 488 nm and 510–535 nm, respectively.

### Flow Cytometry Detection

The number of yeast cells displaying *Bm*AChE was determined using a flow cytometer (BD FAcscalibur, CA, USA) with a 488 nm excitation wavelength and a 525 nm emission wavelength to estimate the percentage of *Bm*AChE molecules displayed.

### Enzyme Activity Determination of the Displayed *Bm*AChE

The activity of the displayed *Bm*AChE was evaluated spectrophotometrically at 405 nm according to Ellman *et al* [25]. using the substrate ATC and the chromogenic reagent DTNB. A cell suspension (100 µL) of transformed *P. pastoris* was centrifuged at 8000×g for 2 min and the cells were weighed. The collected cells (approximately 1.03×10^7^ cells as determined by a hemocytometer) were washed three times with potassium phosphate buffer (3.075 mL of a 1 M K_2_HPO_4_ solution combined with 1.925 mL of a 1 M KH_2_PO_4_ solution (pH 7.0)) and re-suspended in 780 µL potassium phosphate buffer (pH 7.0). The enzymatic reaction was activated by consecutively adding 100 µL of 1 mM ATC and 7.8 mM DTNB. The reaction mixture was incubated at room temperature for 5 min and stopped with 20 µL of 1×10^−7^ M eserine. After centrifugation of the reaction mixture, the supernatant was used to measure the OD at 405 nm with an ELISA reader (Multiscan MK3, Labsystem Co., Finland). One unit of AChE activity was defined as the amount of enzyme hydrolyzing 1 mmol of ATC in 1 min with 1 g of wet cells.

### Inhibition of Displayed *Bm*AChE

Inhibition of the displayed *Bm*AChE was carried out in the presence of eserine [26], a well-known AChE inhibitor previously employed to study the enzyme. Transformants were inoculated on MM-B agar plates (1.34% YNB (v/v), 0.00004% biotin, 1% methanol, 100 mM potassium phosphate buffer (pH 7.0) and 1.8% agarose) at 28°C for 3 d. Then 10 µL of 50 mM potassium phosphate buffer (pH 7.0) was placed on one GS115 colony (negative control) and on one GS115/pPIC9K-*bmace-AGα1* colony (positive control), and 5 µL of 50 mM potassium phosphate buffer (pH 7.0) and 5 µL of different concentrations of eserine (10^−2^–10^−9^ M) were placed on 8 other positive colonies. Following a 10 min reaction period, 3 µL of 10 mM ATC and 3 µL of 7.8 mM DTNB were added to each colony, and the colony color was observed after 10 min at 37°C. Also, an inhibition study of the displayed *Bm*AChE was performed according to Ellman's method [25] using the yeast cell suspension and different concentrations of eserine.

### Detection of CB and OP Pesticides Using the Displayed *Bm*AChE

Five CB pesticides (carbofuran, carbosulfan, isoprocarb, methiocarb and methomyl) and seven OP pesticides (dichlorphos, dimethoate, isocarbophos, malathion, methamidophos, parathion and trichlorphon) were tested (Figure 1). The induced cell suspension was centrifuged at 8000×g for 2 min, re-suspended and adjusted to an OD_600_ of 2 using 50 mM potassium phosphate buffer (pH 7.0). A volume of 120 µL transformed *P. pastoris* cell suspension (approximately 0.25×10^7^) was mixed with the same volume but different concentrations of CB and OP pesticides. After a 5 min incubation at room temperature, 30 µL of 10 mM ATC and 7.8 mM DTNB were consecutively added, and 20 µL of 1×10^−7^ M eserine was added to stop the reaction. The reaction mixture was centrifuged at 8000×g for 2 min and the suspension was removed to microlon plates. The activity of AChE was measured using the multilabel counter at 405 nm. The median inhibition concentration (IC_50_) for each compound was calculated based on the Log-dose versus probit regression [27]. The lowest concentration that could be detected was measured according to the Inhibition Rate (B/B_0_).

## Results and Discussion

### Construction of the *Bm*AChE Yeast Surface Display System Using *P. pastoris*

**Figure 2:**
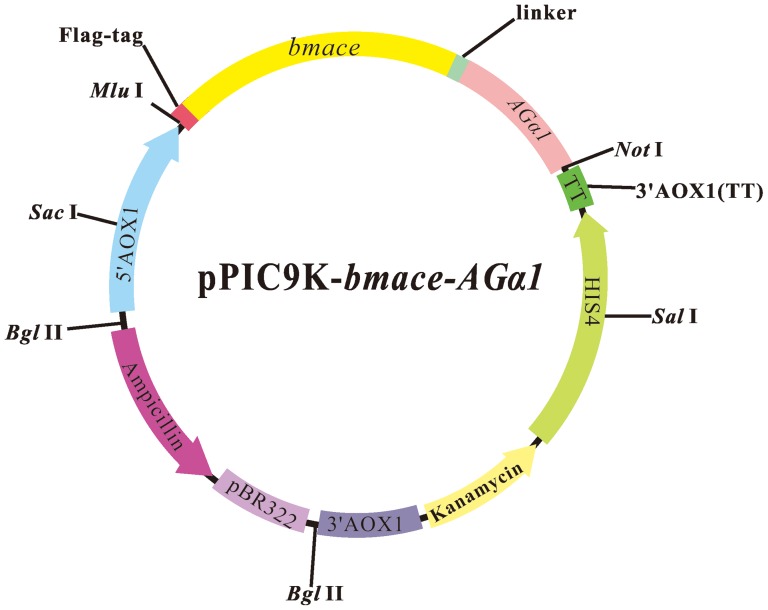
Construction of the *Bm*AChE-display with the *P. pastoris* expression system based on α-agglutinin. ****** The *bmace* and *AGα1* DNA segments were spliced using overlap extension PCR to assemble the *bmace-AGα1* gene and inserted into pPIC9K for the pPIC9K-*bmace-AGα1* construction.

The plasmid for surface display of *Bm*AChE was constructed as shown in Fig. 2. The amplification of *bmace* generated an approximate 1900 bp DNA fragment, while the *AGα1* gene generated an expected 1000 bp fragment. PCR amplification of the assembled *bmace-AGα1* gene produced an expected 2900 bp fragment (Figure S1, see supplementary data for the nucleic acid sequence in the Supporting Information). The *bmace-AGα1* gene with a FLAG tag (eight amino acids) at the *N*-terminus of AChE was subcloned into the expression vector pPIC9K. The results from sequencing indicated the recombinant plasmid pPIC9K-*bmace-AGα1* had been successfully constructed. PCR amplification using AOX-F and AOX-R primers (Table 1) with the genome of the selected transformants as template produced the 2900 bp amplified fragment, indicating that the constructed vectors were integrated into the genome of *P. pastoris* GS115.

### Characterization of the Displayed *Bm*AChE by Fluorescence Microscopy and Flow Cytometry

**Figure 3:**
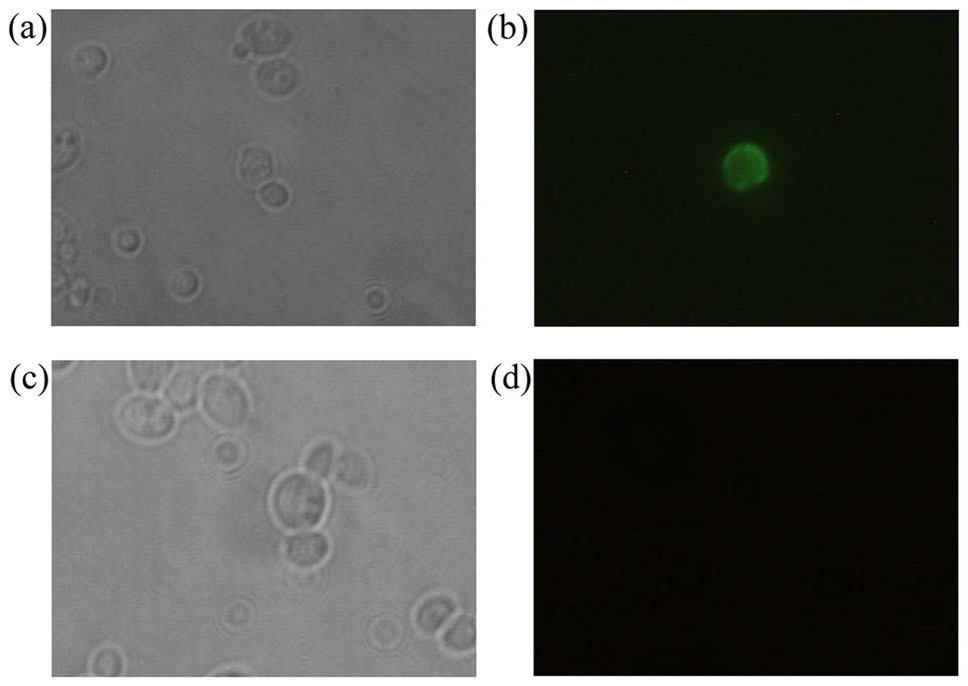
Fluorescence microscopy assay of recombinant *P. pastoris* cells displaying *Bm*AChE: The fluorescence at 519 nm emitted with excitation at 495 nm was observed by fluorescence microscopy. (a) and (c), phase micrographs of recombinant yeast cells; (b) and (d), fluorescent micrographs of recombinant yeast cells. GS115/pPIC9K-*bmace-AGα1* (a, b); GS115 as a control (c, d).

The display of *Bm*AChE on the yeast cell surface was evaluated by immunofluorescence microscopy. Fluorescence was observed on the cell surface of the pPIC9K-*bmace-AGα1* transformant strains using a fluorescence microscope, and fluorescence was not observed from the control cells (Fig. 3). The images demonstrated that the *bmace-AGα1* fusion protein was anchored on the *P. pastoris* surface.

**Figure 4:**
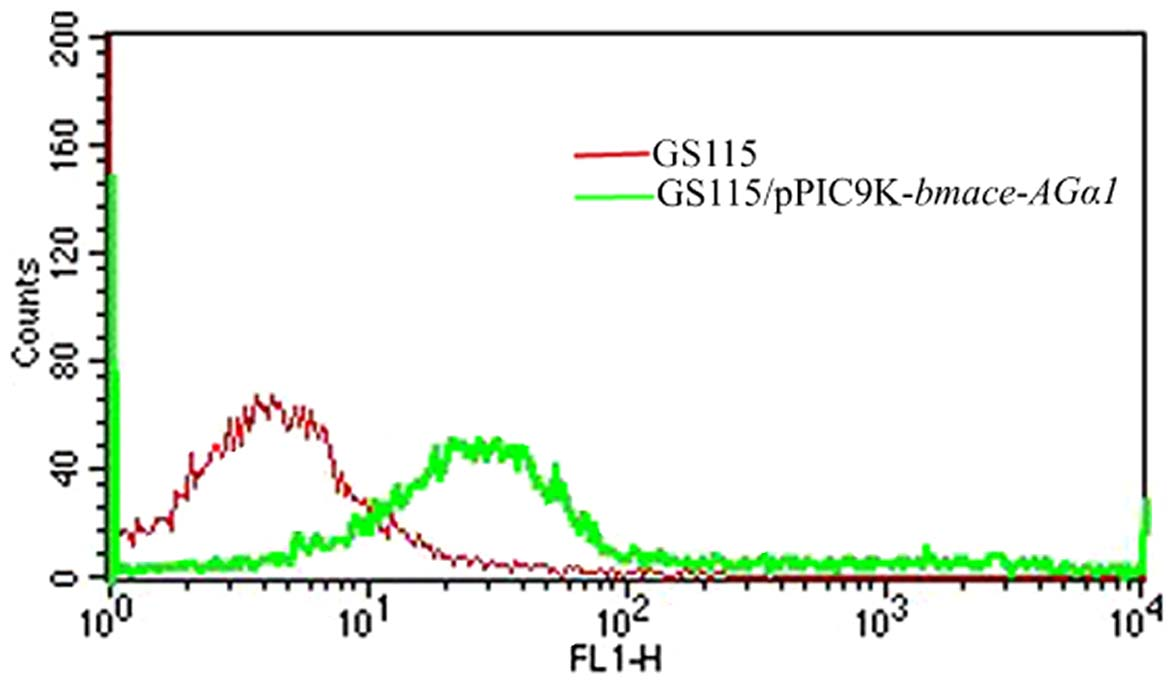
Flow cytometry detection of *Bm*AChE displayed on the recombinant yeast surface.

The expression of the *Bm*AChE fusion protein on the surface of *P. pastoris* was further analyzed by indirect immunofluorescence labeling using flow cytometry (Fig. 4). A difference was detected in the amounts of *Bm*AChE-α-agglutinin fusion protein expression obtained from *P. pastoris* pPIC9K-*bmace-AGα1* transformants. Fluorescence was detected in about 25% of the constructed cells. These studies confirmed that *Bm*AChE was displayed on the cell surface of *P. pastoris*.

### Enzyme Activity Determination Using Displayed *Bm*AChE

AChE activity was measured based on the Ellman method [25] using ATC and DTNB. The hydrolytic activity of the *Bm*AChE enzymes displayed on the surface of the cells was 787.7 U/g (wet cell weight) after being induced with methanol for 4 days at 28°C.

### The inhibition analysis of displayed *Bm*AChE

**Figure 5:**
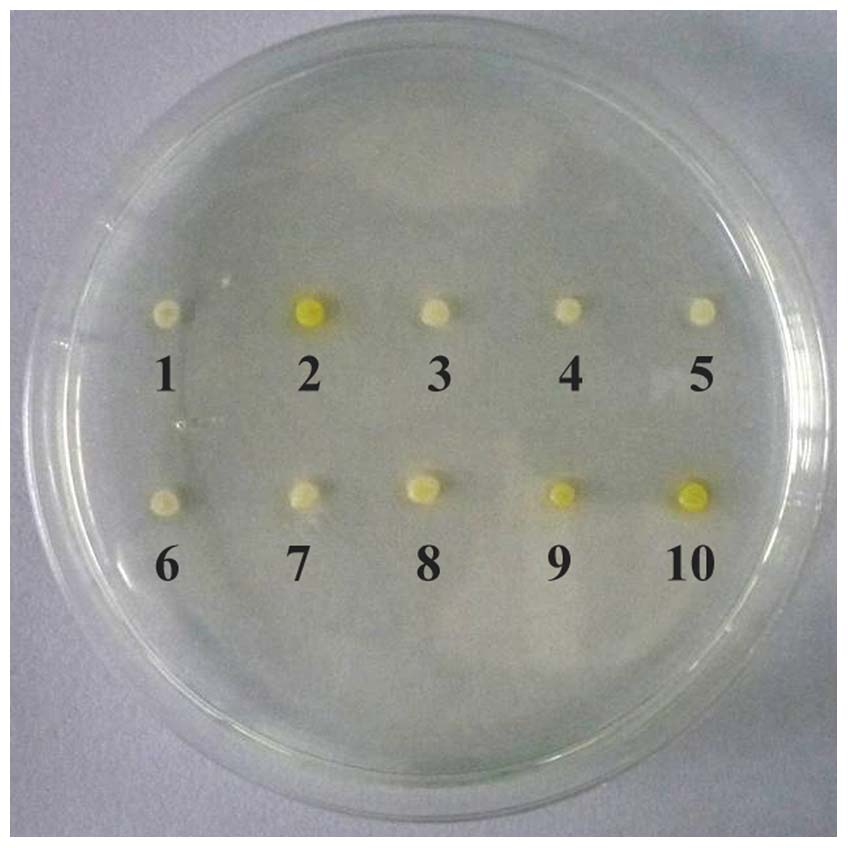
*P. pastoris* display of *Bm*AChE on an MM-B plate: 1, GS115; 2, GS115/pPIC9K-*bmace-AGα1*; 3–10, GS115/pPIC9K-*bmace-AGα1* inhibited by different concentrations of eserine (clone 3: 10^−2^ M, clone 4: 10^−3^ M, clone 5: 10^−4^ M, clone 6: 10^−5^ M, clone 7: 10^−6^ M, clone 8: 10^−7^ M, clone 9: 10^−8^ M, clone 10: 10^−9^ M).

**Figure 6:**
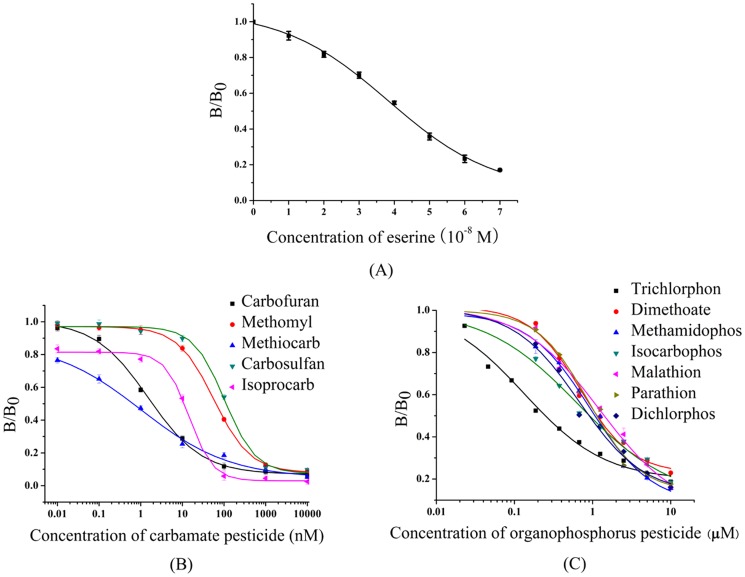
Inhibition curve of displayed *Bm*AChE. (A), inhibition curve of displayed *Bm*AChE for eserine; (B), inhibition curve of displayed *Bm*AChE for CB pesticide (n = 3); and (C), inhibition curve of displayed *Bm*AChE for OP pesticides (n = 3). B, the average absorbance at the indicated concentrations; B_0_, the average absorbance at zero concentration. The data were fitted with a four-parameter-logistic equation to calculate the IC_50_ using OriginPro 7.5 software. The data points are mean values and the errors observed from triplicate determinations.

The inhibition characteristics of eserine, the AChE specific inhibitor, were performed on MM-B plates. As shown in Fig. 5, the color of the original *P. pastoris* GS115 colonies was white (Fig. 5, colony 1), while the color of the transformed *P. pastoris* GS115/pPIC9K-*bmace-AGα1* colonies was yellow (Fig. 5, colony 2). When the concentration of eserine was between 10^−7^–10^−9^ M, the color of the pPIC9K-*bmace-AGα1* colonies gradually turned yellow (Fig. 5, colony 8–10). When the concentration of eserine was 10^−9^ M (Fig. 5, colony 10), the color was close to the positive control (Fig. 5, colony 2). Based on colony color, the inhibition characteristics of eserine for displayed *Bm*AChE were estimated for a concentration series of eserine solutions (1×10^−8^, 2×10^−8^, 3×10^−8^, 4×10^−8^, 5×10^−8^, 6×10^−8^ and 7×10^−8^). The B/B_0_ decreased with increasing eserine concentrations (Fig. 6 (A)). The IC_50_ of *Bm*AChE was 4.17×10^−8^ M. The results showed that the recombinant *Bm*AChE that was displayed on the yeast surface exhibited high-sensitivity to eserine. Compared with the *Bm*AChE expressed in *Trichoplusia ni* (BTI-Tn-5B1-4) cells [28], the sensitivity for eserine in our report was at about the same level, which indicated that *Bm*AChE retained its natural activity after being displayed on the cell surface.

### Detection of CB and OP Pesticides Using Displayed *Bm*AChE

The displayed *Bm*AChE enzyme was used to detect five CB and seven OP pesticides. Measurement of enzyme activity and inhibition studies were performed as described in the experimental section. The inhibition of *Bm*AChE with CB pesticides is shown in Fig. 6 (B). An IC_50_ of 1.92×10^−9^ M was obtained with carbofuran, while an IC_50_ of 1.13×10^−7^ M was obtained with carbosulfan, 1.11×10^−8^ M with isoprocarb, 6.58×10^−8^ M with methiocarb and 6.41×10^−8^ M with methomyl. Inhibition of *Bm*AChE with seven OP pesticides is shown in Fig. 6 (C) and Table 2. Among them, trichlorphon showed the highest inhibitory effect on *Bm*AChE activity with an IC_50_ of 2.40×10^−7^ M and a limit of detection of 3.89×10^−8^ M. The maximum European Union (EU) residue limit was recently set at 0.01 mg/kg (approximately 4.0×10^−8^ M) for pesticide residues in all agricultural products for food or animal feed [29]. Therefore, the activity of the displayed *Bm*AChE has sufficient sensitivity for the determination of most of the selected CB and OP pesticides. As seen in Table 2, for all five tested CB pesticides (carbofuran, carbosulfan, isoprocarb, methiocarb and methomyl), the sensitivity values of the displayed *Bm*AChE were better than those of the common housefly (*Musca domestica*) and those of the common fruit fly (*Drosophila melanogaster*) AChEs. In addition, the sensitivity of our displayed *Bm*AChE for the representative OP pesticides (dimethoate, isocarbophos and trichlorphon) is much better than the housefly AChE [30]. For dichlorphos, the sensitivity of the displayed *Bm*AChE was at the same level as with the *Drosophila melanogaster* AChE [31], but a little less than that of the *Bombyx mandarina* AChE [32]. Further experimental optimization of the *P. pastoris* displayed *Bm*AChE enzyme is expected to meet or exceed the pesticide detection requirements and the displayed *Bm*AChE enzyme will be used for routine monitoring of CB and OP pesticides.

**Table 2:** The median inhibition concentration (IC_50_) of displayed *Bm*AChE for five CB and seven OP pesticides.

Analytes	Displayed *Bm*AChE	Expressed housefly AChE [30]	Expressed *Drosophila melanogaster* AChE [31]	Expressed *Bombyx mandarina* AChE [32]
	IC_50_ (M)	IC_50_ (M)	IC_50_ (M)	IC_50_ (M)
Carbofuran	1.92×10^−9^	1.85×10^−3^	1.02×10^−6^	–
Carbosulfan	1.13×10^−7^	–	–	–
Isoprocarb	1.11×10^−8^	–	–	7.50×10^−7^
Methiocarb	6.58×10^−8^	–	–	–
Methomyl	6.41×10^−8^	3.67×10^−2^	–	6.00×10^−6^
	IC_50_ (M)	IC_50_ (M)	IC_50_ (M)	IC_50_ (M)
Trichlorphon	2.40×10^−7^	6.05×10^−3^	–	–
Dimethoate	1.14×10^−6^	7.74×10^−2^	–	–
Methamidophos	1.07×10^−6^	–	–	–
Isocarbophos	9.10×10^−7^	7.10×10^−2^	–	–
Malathion	1.41×10^−6^	–	–	–
Parathion	1.13×10^−6^	–	–	–
Dichlorphos	9.03×10^−7^	4.87×10^−4^	–	2.40×10^−7^

Analytical equipment-based methods typically used for the analysis of pesticides are not practicle enough to be used for simple, fast detection of large numbers of samples. Rapid assays using AChE-based methods have been proposed as an efficient and rapid method for the detection of pesticides, especially in many Asian markets [5]. Until now, most of the AChE enzymes used for the detection of pesticides have been extracted from fish and insect heads [33], [34], requiring much preparation time, resulting in high costs for enzyme purification. However, the yeast-display technology has provided an alternative means for engineering a low-cost AChE enzyme with desirable activity and the developed cells can be immobilized by chemical methods or with physical methods for development of whole-cell biosensors [35]. Also, the yeast expression system is capable of folding and glycosylating heterologous eukaryotic proteins [36], [37]. In particular, *P. pastoris* also has the advantage of high-density cultivation in inexpensive medium compared with other yeasts [38]. Therefore, the displayed AChE on the cell surface of *P. pastoris* potentially has many benefits and practical applications for pesticide detection.

AChE has been most often used for the detection of pesticides because of its broad-substrate specificity. In this study the AChE gene from *Bombyx mori* was cloned from a constructed vector and a *P. pastoris* cell surface display system was developed for the first time. The surface-displayed *Bm*AChE was evaluated with eserine, and with CB and OP pesticides. The results demonstrated that the displayed *Bm*AChE was bioactive and highly sensitive to CB and OP pesticides. The recombinant *Bm*AChE surface-display can be used for detection of pesticide residues in AChE-based screening methods.

## Supporting Information

Figure S1 **Nucleic acid sequence of the 2900 bp fragment.**
(PDF)
