# Supplementary material for: Surface Display and Bioactivity of Bombyx mori Acetylcholinesterase on Pichia pastoris
Source: PLoS One. 2013 Aug 5;8(8):e70451. doi: 10.1371/journal.pone.0070451 (PMC3734245; doi:10.1371/journal.pone.0070451)
Supplement: Figure S1 — Nucleic acid sequence of the 2900 bp fragment. (PDF) [file pone.0070451.s001.pdf]

acgcgtgactacaaggacgacgacgacaagcgatcttgggccaatcaccatgataccacaacatctaca
Mlu I
acacaaactaccccaacgacaagtccggtaccaaaaatatccacaacgatccacttattgtcgaaacaaa
gagcggtctcatcaaaggatacgcaaaaactgtaatggacgcgaggtacacattttacgggtatcccgt
ttgcgaaacctccattaggaccccctgagattccgtaaaccggtaccaatcgagccatggcatggcgtgctt
gaagcaaacttaatgccaaacagttgctatcaagaacgctacgaatatttcccaggtttcgaaggagaaga
aatgtggaatccaaatactaatatatcagaagattgcctttatttgaatatttgggtaccacagcacttac
gagttcgtcaccatcaagataaaccactcgccgaaagacctaaagtgccgattcttgtgtggatttacggc
ggtggctacatgagtggcgcggctacacttgacctatataaagcagatataatggcatctacaagcgacgt
aatagtggcttctatgcaatacagggttggtgcatttggattttatatttgaataaatatttttctccgg
gtagtgaagaagctcctggaaatatgggtttatgggatcaacaactcgctattcgttggataaaagagaac
gctcgtgcttttggaggagaccctgaactcattacgctgttcggggaatctgccggtggcggtagtgtaag
ccttcatatgctatcacctgaaatgaaaggattgtttaaaagaggtatattgcaatcaggaacgttgaatg
caccttggagttggatgactggagaaagagctcaagatattggaaaagtattaattgatgactgtaactgc
aacagtagtcttttagccaaggatcctagtctcgtaatggattgcatgcgtggagttgacgctaaaacgat
ttctgtccagcaatggaattcttatactggaattttgggttttccgtccgcacctacggttgatggtattt
ttttgccaaaagatcctgataccatgatgaaggaaggaaatttccataatagtgaagtgctacttggcagt
aaccaagacgaaggacatatttttttgctgtacgacttcctggattatttcgaaaaggatgggcctagttt
tcttcagagggagaaatttctcgaaatcgttgacactattttcaaggacttttctaaaattaaaagagaag
ccattgtgttccagtatacagattgggaagagatcaccgacggatatttgaaccagaagatgatagctgat
gtcgtaggagactacttcttcgtatgccccactaactacttcgccgaaatacttgccgacgctggtgtcga
tgtttactattactattttactcatcgtaccagcacaagtctctggggagaatggatgggcgtgatgcatg
gtgacgaaatggaatatgtttttggacatcccttgaacatgtcccttcagtaccattcccgggagcgtgat
ttagcagcacacattatgcagtctttcacacagtttgctcttaccggaaaacctcacaagcctgacgagaa
gtggcctctgtactcccggtcttcgcctcattactacacatacacggcggtgggtccaagcggtccagctg
gaccccgcggcccgcgtgcctccgcttgcgctttctggaacgatttcttgaacaaacttaacgagttggag
cgtgtaccgtgtgacggcgccgtgaccggtccttacagcagtggtggaggcggttcaggcggaggtggctc
tggcggtggcggatcgaacctcggtacagctagcgccaaaagctcttttatctcaaccactactactgatt
taacaagtataaacactagtgcgtattccactggatccatttccacagtagaaacaggcaatcgaactaca
tcagaagtgatcagccatgtggtgactaccagcacaaaactgtctccaactgctactaccagcctgacaat
tgcacaaaccagtatctattctactgactcaaatatcacagtaggaacagatattcacaccacatcagaag
tgattagtgatgtggaaaccattagcagagaaacagcttcgaccgttgtagccgctccaacctcaacaact
ggatggacaggcgctatgaatacttacatctcgcaatttacatcctcttctttcgcaacaatcaacagcac
accaataatctcttcatcagcagtatttgaaacctcagatgcttcaattgtcaatgtgcacactgaaaata
tcacgaatactgctgctgttccatctgaagagcccacttttgtaaatgccacgagaaactccttaaattcc
ttctgcagcagcaaacagccatccagtccctcatcttatacgtcttccccactcgtatcgtccctctccgt
aagcaaaacattactaagcaccagttttacgccttctgtgccaacatctaatacatatatcaaaacgaaaa
atacgggttactttgagcacacggctttgacaacatcttcagttggccttaattcttttagtgaaacagca
gtctcatctcagggaacgaaaattgacacctttttagtgtcatccttgatcgcatatccttcttctgcatc
aggaagccaattgtccggtatccaacagaatttcacatcaacttctctcatgatttcaacctatgaaggta
aagcgtctatatttttctcagctgagctcggttcgatcatttttctgcttttgtcgtacctgctattcgcg
gccgc
Not I
